# Assessment of herbal drugs for promising anti-*Candida* activity

**DOI:** 10.1186/s12906-017-1760-x

**Published:** 2017-05-08

**Authors:** Sameh S. M. Soliman, Mohammad H. Semreen, Ali A. El-Keblawy, Arbab Abdullah, Priya Uppuluri, Ashraf S. Ibrahim

**Affiliations:** 1 Department of Medicinal Chemistry, College of Pharmacy, University of Sharjah, Sharjah, PO Box 27272, United Arab Emirates; 2 Sharjah Institute for Medical Research, University of Sharjah, Sharjah, United Arab Emirates; 3 Department of Applied Biology, University of Sharjah, Sharjah, United Arab Emirates; 4 University Hospital Sharjah, Sharjah, United Arab Emirates; 5 Division of Infectious Diseases, Los Angeles Biomedical Research Institute, Harbor-UCLA Medical Center, Torrance, CA USA; 6 David Geffen School of Medicine at UCLA, Los Angeles, CA USA; 7 Permanent address: Department of Pharmacognosy, Faculty of Pharmacy, University of Zagazig, Zagazig, Egypt

**Keywords:** *Candida albicans*, Antimicrobial, Medicinal plant, Toxicity, Activity, Biofilm

## Abstract

**Background:**

Microbial infections are diverse and cause serious human diseases. *Candida albicans* infections are serious healthcare-related infections that are complicated by its morphological switching from yeast to hyphae, resistant biofilm formation and mixed infections with bacteria. Due to the increase in drug resistance to currently used antimicrobial agents and the presence of undesirable side effects, the need for safe and effective novel therapies is important. Compounds derived from plants are known for their medicinal properties including antimicrobial activities. The purpose of the study was to compare and evaluate the anti-*Candida* activities of several medicinal plants in order for the selection of a herbal drug for human use as effective antimicrobial. The selection was taking into considerations two important parameters; parameters related to the selected drug including activity, stability, solubility and toxicity and parameters related to the pathogen including its different dynamic growth and its accompanied secondary bacterial infections.

**Methods:**

Seven different plants including *Avicennia marina* (*Qurm*), *Fagonia indica* (*Shoka’a*), *Lawsania inermis* (*Henna*), *Portulaca oleracea* (*Baq’lah*), *Salvadora persica* (*Souwak*), *Ziziphus spina- Christi* (*Sidr*) and *Asphodelus tenuifolius* (*Kufer*) were ground and extracted with ethanol. The ethanol extracts were evaporated and the residual extract dissolved in water prior to testing against *Candida albicans* in its different morphologies. The antibacterial and cytotoxic effects of the plants extracts were also tested.

**Results:**

Out of the seven tested plants, *L. inermis* and *P. oleracea* showed significant anti-*Candida* activity with MIC ~10 μg/mL. Furthermore, both plant extracts were able to inhibit *C. albicans* growth at its dynamic growth phases including biofilm formation and age resistance. Accompanied secondary bacterial infections can complicate *Candida* pathogenesis. *L. inermis* and *P. oleracea* extracts showed effective antibacterial activities against *S. aureus*, *P. aeruginosa*, *E. coli*, and the multidrug resistant (MDR) *A. baumannii* and *Klebsiella pneumoniae*. Both extracts showed no toxicity when measured at their MIC on human erythrocytes.

**Conclusion:**

The results from this study suggested that *L. inermis* and *P. oleracea* extracts and/or their chemicals are likely to be promising drugs for human use against *C. albicans* and MDR bacteria.

## Background

The incidence of microbial infections has increased worldwide, in particular in the healthcare settings [1]. One of the most effective strategies to deal with infections has been the use of antimicrobials in prophylaxis or as therapy. However the fast and widespread incidents of drug resistant among pathogenic microorganisms [2, 3], necessitates the constant search for and development of new antibiotics with novel mechanisms of action [4, 5]. The processes of searching for new effective drugs are more complicated for fungal pathogens given the structural similarities between eukaryotes and mammalian cells which often result in effective but toxic drugs [6, 7].

*Candida* is one of the most common human fungal pathogens [8] and represents the most important cause of opportunistic mycoses worldwide [9]. *Candida* is known as a major cause of healthcare-related infections among both immunosuppressed and immunocompetent hosts [10]. It is capable of causing both local and hematogenously disseminated infections [11]. The frequency of healthcare-related candidemia increased dramatically over the last decades and it is now considered as one of the most common bloodstream infections in the intensive care units (ICU) [11, 12]. Despite the increase in *Candida* infections due to non-*albicans* species, *C. albicans* remains the main causative agent of candidemia worldwide [12]. Transplantation, immunosuppression, the use of infected devices including catheters and prolonged hospitalization increase the prevalence of invasive candidiasis [13].

The ability of *C. albicans* to switch from yeast to hyphae is recognized as a virulence factor that enables the organism to invade host tissues [14]. Furthermore, *Candida spp*. produce biofilms on synthetic materials [15]. *Candida* biofilms develop when organisms adhere to a surface allowing the growth of extensive amount of hyphae and produce extracellular polymers that provide a structural matrix to facilitate further adhesion. Biofilms provide a safe haven for *Candida,* facilitate drug resistance, and acts as a sources for chronic infections [16]. Catheter-related microbial biofilms are associated with 90% of *Candida* infections and considered as the major cause of morbidity and mortality among hospitalized patients [17].

Plants have been used in traditional herbal medicine for many years [18]. In some parts of the world, plants and herbs are still the primary source of remedies used in treating diseases [19]. For instance, several plant extracts have been reported to have anti-*Candida* activities including *Allium sativum* (*Garlic*) [20, 21], Berberine-containing herbs [22, 23], *Cinnamomum verum* (*Cinnamon*) and *Origanum vulgare* (*Oregano*) [24–26]. Other plants [27] including *Avicennia marina* (*Qurm*) [28], *Fagonia indica* (*Shoka’a*) [28], *Lawsania inermis* (*Henna*) [29], *Portulaca oleracea* (*Baq’lah*) [30], *Salvadora persica* (*Souwak*) [31–33] and *Ziziphus spina- Christi* (*Sidr*) [34] were also investigated for their antimicrobial activities. However, studies on their anti-*Candida* activities are still in their infancy. Moreover, none of these plant extracts have been approved by regulatory agency for human use either because of lack of information regarding their efficacy/toxicity and/or lack of defined chemical structures. Here, in a preliminary investigation, we evaluated the in vitro activity of seven different plants native to United Arab Emirates (U.A.E.) against healthcare-related pathogens with emphasis on *C. albicans.* Our ultimate goal is to identify novel drugs with significant activity against *Candida Spp.* and MDR bacteria expected to complicate *Candida* infections. Thus it can be defined in their efficacy and toxicity profiles prior to determining their mechanism of action to facilitate their use and evaluation in clinical trials.

## Methods

### Plant samples and extraction procedure

Plants were collected from different locations within the city of Sharjah, U.A.E. on April, 2016 as indicated in Table 1. The plants were taxonomically identified by Dr. Ali El-Keblawy at the Department of Applied Biology, University of Sharjah and voucher specimens were deposited at the University of Sharjah Herbarium on April 2016. The fresh aerial parts of the plants were cut into small sections and ground to very fine paste/powder. The paste/powder was extracted either with ethyl acetate or 95% ethanol three times followed by filtration. The organic solvent extracts were combined separately and evaporated using rotary evaporator at 50 °C till dryness. The residual extract either used directly or left at room (~25 °C) or ~4 °C temperatures for 4 months. The residual extracts were dissolved in sterile water prior to antimicrobial testing and in PBS washing buffer prior to toxicity testing.

**Table 1:** Scientific, traditional names and collecting locations of medicinal plants under study

Plant Scientific Name	Plant Traditional Name	Location
*Avicennia marina*	*Qurm*	Wadi Shawka, Sharjah
*Fagonia indica*	*Shoka’a*	Al Dhaid Bridge sharjah
*Lawsania inermis*	*Henna*	Wadi Shawka, Sharjah
*Portulaca oleracea*	*Baq’lah*	Wadi Shawka, Sharjah
*Salvadora persica*	*Souwak*	Al Dhaid bridge, Sharjah
*Ziziphus spina- Christi*	*Sidr*	Wadi Shawka, Sharjah
*Asphodelus tenuifolius*	*Kufer*	Wadi Shawka, Sharjah

### Studying the anti-*Candida* and anti-bacterial activities of plant extracts

The antimicrobial activity of each plant extract was studied against *C. albicans* (SC5314) and bacteria strains, namely: *S. aureus*, *P. aeruginosa*, *E. coli*, and the multidrug resistant (MDR) *A. baumannii* and *Klebsiella pneumoniae*. All bacterial strains are clinical isolates from patients who were seen at Harbor-UCLA Medical Center, Torrance, CA, U.S.A. The antimicrobial activities of all plant extracts were tested either by disc diffusion assay, in liquid media and by measuring the minimum inhibitory concentration (MIC).

#### Determination of the antimicrobial activity of plant extracts on agar plates and culture broth media

The antimicrobial activity of plant extracts on agar plates, liquid broth media and MIC were measured according to a modified version of Clinical and Laboratory Standards Institute (CLSI) [35]. Briefly, 0.1 mL containing 10^5^ CFU /mL was spread on Luria-Bertani (LB) agar plates [36]. The plates were then incubated at 37 °C with filter discs (8 mm diameter) saturated with different dilutions of plant extracts (25, 50 and 100 μg/mL) for 1, 2 and 3 days. The inhibition zones (mm) were measured by determining the diameter of the clear area. Similarly, the activity in liquid media was measured by incubating the aforementioned concentrations of plant extracts into LB broth media inoculated with 10^5^ CFU/mL in 24-well microplates at 37 °C for 1, 2 and 3 days. For the MICs, different concentrations (1, 2.5, 5, 10, 25, 50, and 100 μg/mL) of plant extracts were added to LB media inoculated with 10^5^ CFU/mL in 96-well microplates for 24 h at 37 °C and the lowest concentration of plant extracts that prevented microbial growth (showed no turbidity) was measured by microplate reader (DYNEX technologies) at OD_600_. Each test was performed in triplicate. Ketoconazole, colistin and vancomycin were employed as positive controls against *Candida*, Gram negative bacteria and Gram positive bacteria, respectively. Cultures without plant extracts or antimicrobials were employed as negative control.

The total activity of each plant was calculated according to the following formula [37]. “Total activity (mL/g) = Amount extracted from 1 g (mg) / MIC (mg/mL)”.

The minimum fungicidal concentrations (MFC) [38] of both *L. inermis* and *P. oleracea* alcoholic extracts were measured by taking a loop full from *C. albicans* culture broth treated with 10, 25 and 50 μg/mL and sub-cultured on LB nutrient agar plates at 37 °C for 24 h. Growth of *C. albicans* on solid media indicated that particular concentration of the extract was unable to inhibit the fungal growth.

#### Inhibition of biofilm formation

The MICs for cells forming biofilm were determined by a microtiter plate assay as described previously [39]. Briefly, each well on a 96-well microtiter plate was filled with 100 μL of RPMI-1640 containing 10^6^ *Candida* cells. After 24 h of incubation at 37 °C, the biofilms were washed and exposed to 100 μL of plant extracts at 0.25, 2.5, 12.5 μg/mL, and the plates were incubated for 24 h at 37 °C. The plant extract was removed and the fungal viability was analyzed using 3-(4,5-dimethylthiazol-2-yl)-2,5-diphenyl tetrazolium bromide (MTT) (Sigma) [40] and the final absorbance was measured at 540 nm. The MIC of plant extract caused 50% inhibition of *Candida* biofilm formation was determined by measuring the metabolic activity of biofilm compared to control [40].

#### Susceptibility testing of *Candida* to the antimicrobial activity of plant extracts

A *C. albicans* culture was developed by inoculating LB broth with 10^6^/ mL *C. albicans* for 24 h at 37 °C. After 24 h, the *C. albicans* culture (OD_600_ = 0.9) was then treated separately with the MFC (25 μg/mL) of either *L. inermis* or *P. oleracea* alcoholic extracts or left as control and incubated for another 24 h at 37 °C. A 5 μL of each extract-treated or untreated *Candida* culture used to inoculate fresh antibiotic-free LB broth culture and incubated for 24 h at 37 °C and the OD_600_ was then measured. All experiments were repeated three times.

### Stability testing of plant extracts

Each plant extract was divided into three portions; one left at room temperature (~ 25 °C) for 4 months, another one was refrigerated at ~4 °C for 4 months and a last one used directly once the extraction was done. This was followed by disc diffusion assay of each treatment and the diameter of zone of inhibition (in mm) was read at 24 h.

### Cytotoxicity assay

The cytotoxic assay of the plants extracts was measured as the amount of hemoglobin released by the lysis of human erythrocytes [41, 42]. Briefly, fresh whole blood from healthy individual was collected into heparinized vacutainer from Harbor-UCLA Hospital and 1 mL whole blood was immediately centrifuged at 500 *g* for 10 min using benchtop centrifuge (Eppendorf 5804R Refrigerated Benchtop). The erythrocytes were washed three times with DPBS supplemented with 1 mg/mL bovine serum albumin (BSA) and then re-suspended to 3 × 10^7^ cells/ mL in DPBS. Washed cells (3 × 10^6^ cells per well) were incubated with the total plant extracts dissolved in the washing buffer at different concentrations (ranging from 3.6 to 100 μg/mL) in round-bottomed 96-well plates in a final volume of 200 μL. Washing buffer and 0.1–1% Triton X-100 were used as negative and positive controls, respectively. The plate was incubated at 37 °C for 30 min, followed by 30 min incubation on ice, and the intact cells were precipitated by centrifugation at 500 *g* for 10 min at 4 °C and the supernatants (125 μL) were transferred to a flat-bottom 96-well plate to measure hemoglobin release by absorbance at 405 nm using a microplate reader. The absorbance values for each sample were subtracted from the absorbance value obtained for washing buffer-treated cells and the hemolytic activity (%) was calculated. The experiment was conducted in triplicate and the data was analyzed using two-way analysis of variance (ANOVA).

The 50% cytotoxic concentration (CC_50_) values were calculated as the concentration of plant extract caused 50% hemolysis compared to 100% hemolysis of erythrocytes treated with 1% triton X-100. And selective activities of the extracts were calculated according to the following formula “Selectivity index (SI) = (CC_50_ in mg/mL)/ (MIC in mg/mL)” [43].

### Statistical analysis

The data was collected and graphed using Microsoft Excel^®^. Data was then exported to Graph Pad 5.0 for Windows (GraphPad Software, La Jolla, CA, USA) for statistical analysis. The effects of plant extracts on *C. albicans* inoculated onto solid agar media, liquid broth and during biofilm formation was analyzed using one-way analysis of variance (ANOVA) using Dunnett’s Multiple Comparison Test. *P* value <0.05 was considered as significant.

## Results and discussion

Choosing a medicinal plant to be used as a supplier of antimicrobial drugs is challenging and several issues have to be addressed prior to advancing into clinical trial testing. For example, the efficacy, toxicity and possible kinetics of the drug should be considered.

### Extract selection based on screening for anti-*Candida* activity

In this study, the potentiality of seven medicinal plants including *Avicennia marina*, *Fagonia indica*, *Lawsania inermis*, *Portulaca oleracea*, *Salvadora persica*, *Ziziphus spina- Christi* and *Asphodelus tenuifolius* were compared for their activities against *C. albicans.* The effect of both ethyl acetate and alcoholic (95% ethanol) plants extracts of the aforementioned medicinal plants were tested against wild type *C. albicans* (SC5314) on LB-agar media using disc diffusion assay. Paper discs saturated with plant extracts at 25, 50, and 100 μg/mL were applied on LB solid media streaked with *C. albicans* and incubated at 37 °C for 72 h. *A. tenuifolius*, *S. persica, L. inermis* and *P. oleracea* alcoholic extracts inhibited growth of *C. albicans* after 24 h of incubation (Table 2); However only *L. inermis* and *P. oleracea* alcoholic extracts showed significant (*P* < 0.05) growth inhibition activity up to 72 h (Table 2).

**Table 2:** Inhibition zones diameters (mm) of alcoholic plant extracts against *C. albicans* using disc diffusion assay

	Effect on *C. albicans* streaked on solid LB media (zone of inhibition in mm)								
Days of incubation	Day 1			Day 2			Day 3		
Extract Conc. (μg/mL)	25	50	100	25	50	100	25	50	100
*A. marina*	na	na	na	na	na	na	na	na	na
*F. indica*	na	na	na	na	na	na	na	na	na
L. inermis*	15 ± 0.5	18 ± 0.7	22 ± 1	15 ± 0.2	17 ± 0.5	21 ± 0.4	13 ± 0.5	16 ± 0.5	19 ± 0.3
P. oleracea*	11 ± 1	14 ± 0.6	17 ± 0.5	10 ± 0.2	12 ± 0.3	15 ± 0.2	10 ± 0.2	11 ± 0.1	13 ± 0.2
*S. persica*	10 ± 0.2	12 ± 0.25	17 ± 0.5	na	na	na	na	na	na
*Z. spina- Christi*	na	na	na	na	na	na	na	na	na
*A. tenuifolius*	9 ± 0.1	11 ± 0.09	16 ± 0.5	na	na	na	na	na	na

*na*: no activity; diameter of the paper disc: 8 mm; * Significant difference with *P* value < 0.05 (measured by one-way analysis of variance (ANOVA)). The standard error represents the mean of three replicas

*Candida* infections are complicated by many factors including nutritional conditions, planktonic versus biofilm modes of growth, and the adaptability of the pathogen [44]. All factors together should be considered to provide an effective inhibition of the microbe; so sequential experiments were conducted in order to decide a promising lead extract out of tested plant extracts.

### Extract selection based on differential growth conditions of *Candida*

Since *Candida* shows medium-dependent expression of hyphae specific genes with prominent expression in liquid media compared to solid media [45, 46], all plants extracts under study were evaluated for their ability to inhibit *C. albicans* in liquid LB media. Similar to disc diffusion assay, plant extracts at 25, 50 and 100 μg/mL were added to LB broth media inoculated with *C. albicans* and incubated for 72 h at 37 °C. *A. tenuifolius* and *S. persica* as well as *L. inermis* and *P. oleracea* alcoholic extracts significantly (*P* < 0.05) inhibited growth of *C. albicans* to 24 and 72 h post-incubation, respectively (Table 3). The other plant extracts including *A. marina*, *F. indica* and *Z. spina- Christi* increased the growth of *C. albicans* at lower concentrations, similar to some plant extracts such as green tea leaf extract [47] and cabbage leaf extract [48] that can selectively inhibit and stimulate different microbial growth. All ethyl acetate extracts showed no activity either on solid or liquid media (data not shown).

**Table 3:** The effect of alcoholic plant extracts on the growth of *C. albicans* inoculated into LB broth media

	Effect on *C. albicans* inoculated into LB broth (% growth in relation to negative control)								
Days of incubation	Day 1			Day 2			Day 3		
Extract Conc. (μg/mL)	25	50	100	25	50	100	25	50	100
*A. marina*	200 ± 5.2	100 ± 3.1	70 ± 4.2	100 ± 2.4	100 ± 2.3	100 ± 2.3	100 ± 3.7	100 ± 5.2	100 ± 3.1
*F. indica*	180 ± 0.4	100 ± 3.2	60 ± 3.5	100 ± 0.1	100 ± 3.5	100 ± 4.2	100 ± 4.4	100 ± 5.4	100 ± 3.9
L. inermis*	0 ± 0.2	0 ± 0.4	0 ± 1.2	8 ± 0.4	0 ± 1.0	0 ± 0.5	25 ± 0.2	16 ± 0.4	0 ± 0.4
P. oleracea*	8 ± 0.5	0 ± 0.5	0 ± 1.5	19 ± 0.3	0 ± 0.3	0 ± 0.3	40 ± 0.1	33 ± 0.3	27 ± 0.2
S. persica*	15 ± 0.2	9 ± 0.6	0 ± 0.3	35 ± 0.4	20 ± 0.2	20 ± 0.1	100 ± 0.4	100 ± 1.1	100 ± 0.5
*Z. spina- Christi*	250 ± 2.1	150 ± 0.8	100 ± 0.5	100 ± 0.3	100 ± 2.4	100 ± 0.6	100 ± 1.3	100 ± 0.9	100 ± 0.5
A. tenuifolius*	23 ± 0.5	15 ± 0.3	0 ± 0.1	50 ± 0.5	45 ± 0.2	30 ± 1.2	100 ± 1.2	100 ± 0.4	100 ± 0.6

* Significant difference with *P* value < 0.05 (measured by one-way analysis of variance (ANOVA)). The standard error represents the mean of three replicas. The growth measured by absorbance at OD600 by microplate reader

### Extract selection based on growth complication of *Candida* by morphology changes and biofilm formation

**Fig. 1:**
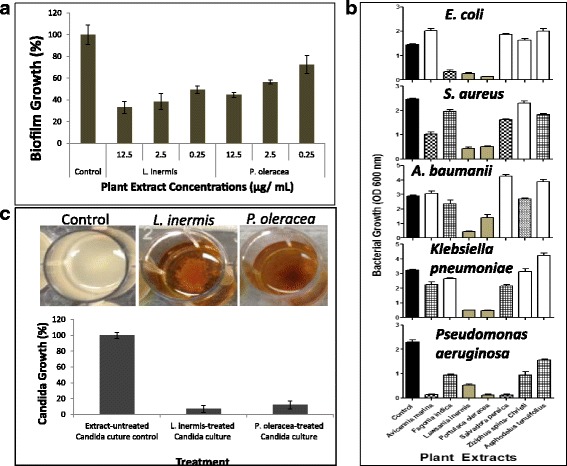
Antimicrobial activities of alcoholic plant extracts. **a** Quantitative microtiter plate assay for biofilm formation using MTT method. The effect of both *L. inermis* and *P. oleracea* plants extracts were tested on *C. albicans* compared to no extract as negative control. **b** Bacterial growth inhibition by crude alcoholic plant extracts. The effect of alcoholic plant extracts on the growth of *E. coli*, *S. aureus*, *Acinetobacter baumanii*, *Klebsiella pneumoniae*, and *Pseudomonas aeruginosa* was tested in 24-well micro-plates. **c** Influence of *L. inermis* and *P. oleracea* alcoholic extracts on 24 h-grwoing *C. albicans* in batch culture. The graph represents the re-inoculation of either alcoholic plant extracts-treated *Candida* or untreated cultures into fresh antibiotic-free media followed by incubation at 37 °C for 24 h. The data was analyzed using one-way analysis of variance (ANOVA) using Dunnett’s Multiple Comparison Test. *P* value < 0.05 was considered as significant. The standard error represents the mean of three replicas

An important feature of *C. albicans* growth is its ability to switch between yeast and hyphae forms [49]. The hyphae form is importantly required for disease progression by invading host cells and causing tissue damage [50, 51], and for formation of biofilm [52]. Because both *L. inermis* and *P. oleracea* showed significant inhibitory effect on *C. albicans* in solid and liquid media, they were tested against biofilm formation. Both alcoholic plant extracts showed significant (*P* value < 0.05) inhibitory effect on *C. albicans* biofilm formation (Fig. 1a) within the range of MIC (Table 4). The MIC of both *L. inermis* and *P. oleracea* were measured to be 10 μg/mL (Table 4) compared to 1 μg/mL ketoconazole (Sigma) as control. And the minimum fungicidal concentrations (MFC) of both *L. inermis* and *P. oleracea* was ≤25 μg/mL.

**Table 4:** Minimum inhibitory concentration (MIC) values in μg/mL of alcoholic plant extracts against *C. albicans* in 24 h incubation period. MIC is the lowest concentration of plant extracts that inhibited microbial growth

	MIC (μg/ mL)					
Microbes	*C. albicans*	*E. coli*	*S. aureus*	*P. aeruginosa*	*A. baumannii*	*K. pneumoniae*
*A. marina*	ND	ND	10 ± 0.8	2.5 ± 0.6	ND	50 ± 0.4
*F. indica*	ND	5 ± 1.3	50 ± 1.6	25 ± 0.4	25 ± 0.9	50 ± 0.7
*L. inermis*	10 ± 1.3	5 ± 0.4	2.5 ± 0.5	5 ± 1.2	2.5 ± 0.5	2.5 ± 0.6
*P. oleracea*	10 ± 0.2	2.5 ± 0.3	5 ± 0.2	2.5 ± 0.4	10 ± 1.2	2.5 ± 0.1
*S. persica*	25 ± 0.5	ND	25 ± 1.2	2.5 ± 0.6	ND	50 ± 1.4
*Z. spina- Christi*	ND	ND	50 ± 0.4	25 ± 1.1	25 ± 0.4	100 ± 0.5
*A. tenuifolius*	50 ± 0.4	ND	25 ± 0.7	50 ± 0.6	ND	ND
Ketoconazole	1 ± 0.25	-	-	-	-	-
Colistin	-	2.5 ± 0.5	-	0.7 ± 0.2	1.25 ± 0.25	10 ± 0.75
Vancomycin	-	-	10 ± 0.5	-	-	-

*ND*: Not determined; The standard error represents the mean of three replicas

### Extract selection based on growth complication of *Candida* by bacterial mixed infections

Another complication with in vivo *Candida* infection is its frequent ability to form mixed infections with bacterial species including *Pseudomonas aeruginosa* usually found in combination in biofilm formation and recovered from patient with lung infection [53], *Staphylococcus aureus* and *Escherichia coli* in inflamed palatal mucosa, enterococci and *Klebsiella* in labial lesion and other infections that can induce life-threatening septicemia [54]. Both *L. inermis* and *P. oleracea* alcoholic extracts showed consistent broad spectrum antibacterial activity to all tested microorganisms including *E. coli*, *S. aureus*, *A. baumanii*, *K. pneumoniae* and *P. aeruginosa* compared to other aforementioned plant extracts (Fig. 1b). Other plant extracts including *A. marina*, *F. indica*, *S. persica*, *Z. spina*, and *A. tenuifolius* showed modest species-specific antibacterial activities (Fig. 1b).

### Extract selection based on complication of *Candida* age and resistance

The relative susceptibility of *C. albicans* to antibiotics is dependent on the age of culture because the culture environment is rapidly changing and the cell populations becomes more physiologically heterogeneous [55] and hence, more resistant with age [56–58]. So it is beneficial to test the effect of both plant extracts on *C. albicans* culture in its stationary phase of growth [59]. A *C. albicans* culture was developed by growing LB broth inoculated with *C. albicans* for 24 h prior to treating separately with *L. inermis* or *P. oleracea* alcoholic extracts. Both extracts caused aggregation and precipitation of the *C. albicans* culture (Fig. 1c). Inoculation of plant extract treated-cultures into fresh antibiotic-free LB broth followed by incubation for 24 h at 37 °C showed >90% inhibition in growth compared to control LB broth that received the same volume of untreated *C. albicans* culture (Fig. 1c). The results indicated that *C. albicans* cultures showed high sensitivity to both plant extracts even at increased growth rate and the effect of the two plant extracts are cidal.

### Selection based on extract stability

Usually antimicrobials are under suspicion of diminishing activities either because of admixture and dispensing to be stored at home or shelf storage before use [60]. The stability during shelf half-life storage of both *L. inermis* and *P. oleracea* alcoholic extracts were tested by storing both plant extracts for 4 months at room temperature (~25 °C) followed by incubation with aforementioned microbes. The results showed that both extracts possess activities similar to those used fresh or stored at 4 °C (data not shown). The results indicated that both plant extracts are stable at wide range of temperatures making them adequate for long storage, and different handling environment. These stability features make both extracts desirable for further development as potential antifungal agents [61].

### Selection based on extract safety

**Fig. 2:**
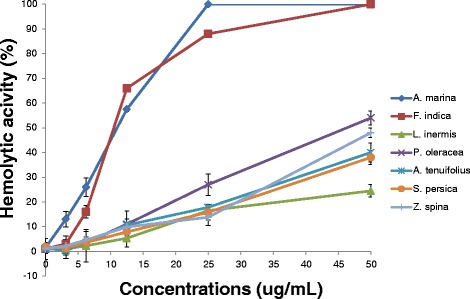
Dose-dependent hemolytic activity of alcoholic plant extracts to human erythrocytes. DPBS-washed erythrocytes (3 × 10^6^ cells per well) were incubated in 96-well plate with the total plant extracts at different concentrations (ranging from 3.6 to 100 μg/mL) at 37 °C for 30 min. The hemoglobin released from lysed erythrocytes was measured using micro-plate reader at 405 nm. The absorbance values for each sample were subtracted from the absorbance value of cells treated only with washing buffer and the hemolytic activity (%) was calculated. The experiment was conducted in triplicate

The adverse drug effects associated with the use of antimicrobials can be of a major concern especially with antifungal agents due to the eukaryotic nature of the organism being targeted. Therefore, it is important to test the toxicity of plant extracts prior to application as antimicrobials. Among the cytotoxicity tests is hemolytic activity assay of human erythrocytes [62]. A cytotoxicity assay was conducted by testing different plant extracts at different concentrations and by using fresh human erythrocytes. The results showed that all plant extracts under study except *A. marina* and *F. indica* are safe and not toxic at a wide range of growth inhibitory concentrations (3–30 μg/mL) (Fig. 2). Both CC50 (cytotoxicity) and selective activity of the plant extracts were measured (Table 5). Our data show that both *L. inermis* and *P. oleracea* exhibit high selective antimicrobial activities. The relatively high selectivity indices of both *L. inermis* and *P. oleracea* indicate that both extracts are likely useful in managing infections due to *C. albicans* and other bacterial infections in humans [43]. The total activity of both *L. inermis* and *P. oleracea* plants were also calculated as 1.7 and 2.1 mL/g, respectively indicative of higher potency of both plants against *C. albicans*. And the results from this research indicate that both *L. inermis* and *P. oleracea* plants could be promising antimicrobials once they promoted for in vivo and clinical studies.

**Table 5:** Selective indices values of alcoholic plant extracts against *C. albicans* and bacterial pathogens

	SI					
Microbes	*C. albicans*	*E. coli*	*S. aureus*	*P. aeruginosa*	*A. baumannii*	*K. pneumoniae*
*A. marina*	0.2	0.13	1.3	5	0.13	0.3
*F. indica*	0.12	2.5	0.25	0.4	0.6	0.21
*L. inermis*	10	20	40	20	40	40
*P. oleracea*	5	20	10	20	5	20
*S. persica*	2.6	0.8	2.6	32	0.8	1.6
*Z. spina- Christi*	0.6	0.6	1	2	3	0.9
*A. tenuifolius*	1.8	0.7	2.3	1.4	0.7	0.7

## Conclusion

Several medicinal plants have been shown to have promising antimicrobial activities in vitro. However, to date there has been little interest in developing these medicinal plants as a source for producing novel drugs against infectious diseases. We show that *L. inermis* and *P. oleracea* plants extracts have promising antimicrobial selectivity against *C. albicans* in its different dynamic forms of growth in vitro. Furthermore, both extracts showed significant antibacterial activity against multidrug resistant bacteria (MDR), that can complicate *Candida* infection through secondary mixed infections. Considering also the lower cytotoxicity and higher selectivity indices, both plant extracts represent promising area of future research that is likely to include in vivo testing, and determination of mechanism of action. Moreover, the active pure compounds from both plant extracts need to be determined which are likely to aid in determining the mechanism of action and the microbial target. Additionally, the ability of both plant extracts at sub-MIC concentrations to modulate the activity of available anti-*Candida* and *Candida* resistance can be addressed in future too. On the other hand, few other plant extracts from this research showed stimulatory effect on *C. albicans* and bacterial growth which can be used to stimulate the growth and detection of difficult-growing beneficial microflora including endophytes.

## Abbreviations

ANOVA: One-way analysis of variance
BSA: Bovine serum albumin
*C. albicans*: *Candida albicans*
CC50: Cytotoxic concentration of the extracts to cause death to 50% of viable cells
CFU: Colony-forming unit
CLSI: Clinical and Laboratory Standards Institute
DPBS: Dulbecco’s phosphate-buffered saline
ICU: Intensive care units
IRB: Institutional review board
LA: Los Angeles
LB: Luria-Bertani
MDR: Multidrug resistant
MFC: Minimum fungicidal concentrations
MIC: Minimum inhibitory concentration
MTT: 3-(4,5-Dimethylthiazol-2-yl)-2,5-diphenyl tetrazolium bromide
OD_600_: Optical density of a sample measured at a wavelength of 600 nm
PBS: Phosphate-buffered saline
*P*-value: Probability value
RPMI: Roswell Park Memorial Institute medium
SI: Selectivity Index
U.A.E.: United Arab Emirates
